# Structure and Dynamics of the gp120 V3 Loop That Confers Noncompetitive Resistance in R5 HIV-1_JR-FL_ to Maraviroc

**DOI:** 10.1371/journal.pone.0065115

**Published:** 2013-06-28

**Authors:** Yuzhe Yuan, Masaru Yokoyama, Yosuke Maeda, Hiromi Terasawa, Shinji Harada, Hironori Sato, Keisuke Yusa

**Affiliations:** 1 Transfusion Transmitted Diseases Center, Institute of Blood Transfusion, Chinese Academy of Medical Science, Chenghua District, Chengdu, Sichuan Province, P. R. China; 2 Pathogen Genomics Center, National Institute of Infectious Diseases, Musashi Murayama, Tokyo, Japan; 3 Department of Medical Virology, Graduate School of Medical Sciences, Kumamoto University, Kumamoto, Japan; 4 Division of Biological Chemistry and Biologicals, National Institute of Health Sciences, Setagaya, Tokyo, Japan; Institut Pasteur, France

## Abstract

Maraviroc, an (HIV-1) entry inhibitor, binds to CCR5 and efficiently prevents R5 human immunodeficiency virus type 1 (HIV-1) from using CCR5 as a coreceptor for entry into CD4^+^ cells. However, HIV-1 can elude maraviroc by using the drug-bound form of CCR5 as a coreceptor. This property is known as noncompetitive resistance. HIV-1_V3-M5_ derived from HIV-1_JR-FLan_ is a noncompetitive-resistant virus that contains five mutations (I304V/F312W/T314A/E317D/I318V) in the gp120 V3 loop alone. To obtain genetic and structural insights into maraviroc resistance in HIV-1, we performed here mutagenesis and computer-assisted structural study. A series of site-directed mutagenesis experiments demonstrated that combinations of V3 mutations are required for HIV-1_JR-FLan_ to replicate in the presence of 1 µM maraviroc, and that a T199K mutation in the C2 region increases viral fitness in combination with V3 mutations. Molecular dynamic (MD) simulations of the gp120 outer domain V3 loop with or without the five mutations showed that the V3 mutations induced (i) changes in V3 configuration on the gp120 outer domain, (ii) reduction of an anti-parallel β-sheet in the V3 stem region, (iii) reduction in fluctuations of the V3 tip and stem regions, and (iv) a shift of the fluctuation site at the V3 base region. These results suggest that the HIV-1 gp120 V3 mutations that confer maraviroc resistance alter structure and dynamics of the V3 loop on the gp120 outer domain, and enable interactions between gp120 and the drug-bound form of CCR5.

## Introduction

Inhibiting the entry of R5 human immunodeficiency virus type 1 (HIV-1) into CCR5^+^/CD4^+^ cells is an effective step in blocking viral replication. An entry inhibitor can bind to CCR5 and prevent R5 HIV-1 from using CCR5 as a coreceptor for entry [1]. Maraviroc, a CCR5 antagonist, has potent *in vitro* and *in vivo* antiviral activity against laboratory strains and clinical isolates [2]–[4]. Maraviroc, approved in 2007, was the first CCR5 antagonist approved by the US Food and Drug Administration and is currently used to treat patients with R5-tropic HIV-1 infections.

Treatment failures can occur because of an increasing number of pre-existing CXCR4-using viruses [5], [6]. Alternatively, escape mutants can evade a CCR5 inhibitor by accumulating multiple mutations in gp120 and/or gp41 without switching their coreceptor usage [7]–[14]. Escape mutants can use the drug-bound form of CCR5 as a coreceptor, a property known as noncompetitive resistance [8], [9], [11]. In noncompetitive-resistant viruses, drug-free CCR5 usage is compatible with the additional ability of drug-bound CCR5 usage. We previously reported that a combination of polymorphic mutations in the gp120 V3 loop can confer noncompetitive resistance in HIV-1_JR-FL_ [15]. One of these viruses, designated HIV-1_V3-M5_, contains a set of five mutations I304V/F312W/T314A/E317D/I318V in the V3 loop (from Cys^293^ to Cys^327^). Most other noncompetitive-resistant viruses contain multiple mutations in the V3 loop [8], [9], [11], although mutations reported till date in the V3 loop are not always common and resistance-associated mutations in the V3 loop were considered to be background dependent. Two elements are involved in gp120 coreceptor binding: (i) the V3 tip for the CCR5 extracellular loop 2 (ECL2) and (ii) the V3 base and stem residues and the V3 base of the gp120 core for the CCR5 N terminus [16]–[19]. Thus, the V3 loop of HIV-1 plays a pivotal role in its interaction with CCR5. However, how the V3 mutations induce maraviroc-resistance without changing coreceptor tropism remains unknown.

Increasing evidence indicates that the protein surface fluctuates in solution, and that such fluctuations play key roles in interactions with other molecules [20] [20], [23]. We previously suggested that the structural dynamics of the HIV-1 gp120 V3 loop play key roles in modulating viral interactions with various molecules, including HIV-1 coreceptors and anti-V3 antibodies [21], [22]. Therefore, it is conceivable that the V3 mutations that cause changes in the structural dynamics of the V3 loop may also be important for viral interactions with the maraviroc and CCR5 complex.

In this study, we examined how the V3 mutations, which conferred maraviroc resistance in HIV-1_JR-FL_, affect the structural dynamics of the V3 loop on the gp120 outer domain. We initially performed extensive mutagenesis on the V3 loop to clarify a genetic basis for maraviroc-resistance of the HIV-1_JR-FL_ strain. These studies demonstrated that combinations of V3 mutations are required to render maraviroc resistance to HIV-1_JR-FL_. Subsequently, we performed MD simulations [23]–[25] of HIV-1_JR-FL_ gp120 outer domains carrying V3 loops with and without the five maraviroc resistance mutations. The results illustrate that at the atomic-level maraviroc resistance mutations affect intrinsic structural properties and motion of the V3 loop on the HIV-1 gp120 outer domain.

## Materials and Methods

### Cells and Viruses

PM1/CCR5 cells were generated from the human CD4^+^ T-cell line PM1 [26] by standard retrovirus-mediated transduction with pG1TKneo-CCR5 [27]. The cells were maintained in RPMI 1640 (Invitrogen) supplemented with 10% heat-inactivated fetal calf serum (FCS; Vitromex). MAGIC-5 cells (HeLa-CD4^+^-CCR5^+^-LTR-b-galactosidase) [28], used as reporter cells for HIV-1 infection, and 293T cells were maintained in Dulbecco's modified Eagle's medium (ICN Biomedicals) supplemented with 10% heat-inactivated FCS. pJR-FL was kindly provided by Prof. Koyanagi (Kyoto University).

### MD simulation

HIV-1 gp120 outer domain structures with various V3 regions were constructed by the homology modeling method, using Molecular Operating Environment (MOE) software v. 2010.10 (Chemical Computing Group Inc., Montreal, Quebec, Canada) [22]. For the modeling template, we used the crystal structure of HIV-1 gp120 containing an entire V3 region at a resolution of 3.30 Å (PDB code: 2QAD) [29]. The 186 amino-terminal and 27 carboxyl-terminal residues were deleted to construct the gp120 outer domain structure. MD simulations were performed using the SANDER module of the AMBER 9 program package [30], the AMBER99SB force field [31], and the TIP3P water model [32]. Bond lengths involving hydrogen were constrained using SHAKE algorithm [32] and the time for all MD simulations was set to 2 fs. A nonbonded cutoff of 12 Å was used. After heating calculations for 20 ps until 310 K using the NVT ensemble, simulations were conducted with the NPT ensemble at 1 atm and 310 K for 20 ns. Superimposition of structures was performed by coordinating the atoms of the amino acids along the β-sheet at the gp120 core. We calculated the root mean square fluctuation (RMSF) to determine the atomic fluctuations along the trajectory broken down by residues during MD simulations. Average structures during the final 10 ns of MD simulations were used as reference structures. RMSFs were calculated using the ptraj module of AMBER 9 [22].

### V3 mutant viruses

V3 mutant proviruses were constructed from pJR-FL_an_. The 176-bp DNA fragments containing single mutations (I304V, F312W, T314A, E317D, or I318V) were subcloned into a cloning vector by overlapping PCR using primers tagged with a mutated tail. The mutation-containing DNA fragments encoding the V3 loop were repeatedly amplified from the cloning vectors using the primers VV-Af (5′-ACAGCTTAAGGAATC TGTAGAAATTAATTG-3′) and VV-Nh (5′-ATTTGCTAGCTATC TGTTTTAAAGTGTCAT-3′). Products were digested with AflII and NheI, subcloned into pCR-SXΔAN, and designated as pCR-SX_1_, pCR-SX_2_, pCR-SX_3_, pCR-SX_4_, and pCR-SX_5_. The *Stu* I–*Xho* I fragment from the plasmids was then subcloned into pJR-FLΔSX that was created by replacing the *Stu* I–*Xho* I fragment of pJR-FL with a linker. The end products were proviral plasmids that were used for transfection for virus production. The procedure described above was repeated for construction of the proviral DNA containing two to four mutations.

For virus preparation, 293T cells (2×10^6^) were transfected with 10 µg of proviral DNA using the calcium phosphate ProFection Mammalian Transfection System (Promega). The supernatant was collected 28 h after transfection, filtered through a 0.22-µm filter (Millipore), and stored at −80°C until further use. The amount of p24 Gag in the supernatant was measured by p24 Gag ELISA (Zeptometrix).

### Viral replication assay

For the viral replication assay, 4×10^4^ PM1/CCR5 cells were infected with 8 ng p24 Gag for 2 h in the presence or absence of 1 µM maraviroc. After washing twice with phosphate-buffered saline (PBS), the infected cells were incubated at 37°C in a 5% CO_2_ atmosphere in the presence or absence of 1 µM maraviroc. On day 6 after infection, the amount of p24 Gag in the supernatant was measured by p24 Gag ELISA (Zeptometrix). Maraviroc was provided by the NIH AIDS Research and Reference Reagent Program, Division of AIDS National Institute of Allergy and Infectious Diseases.

### Determination of drug susceptibility

Drug susceptibilities were determined by the single-round viral entry assay using previously titrated pseudotyped virus preparations with MAGIC-5 cells. In brief, MAGIC-5 cells were plated in 48-well tissue culture plates 1 day before infection. After absorption of the pseudotyped virus for 2 h at 37°C in the presence or absence of 1 µM maraviroc, the cells were washed twice with PBS and further incubated for 48 h in fresh medium in the presence or absence of the inhibitor.

### HIV-1 single-cycle luciferase reporter assay

HIV-1 single-cycle luciferase reporter viruses were produced by cotransfection of 293T cells with pNL–LucR–E^−^ [33] and Env-expressing plasmids pCXN–EnvJR-FLan, pCXN–Env_V3-M5_, pCXN–Env_2345_, pCXN–Env_1345_, pCXN–Env_1245_, pCXN–Env_1235_, or pCXN–Env_1234_. Culture supernatant containing pseudoviruses at a final concentration of 1 ng/ml p24 was added to 1×10^4^ cells/well MAGIC5 cells [28] in a 48-well plate. After 2 h, the cells were washed twice with phosphate-buffered saline (PBS) and firefly luciferase activity was measured 48 h postinfection, according to the manufacturer's directions (Promega).

## Results

### Noncompetitive-resistant virus HIV-1_V3-M5_

**Figure 1:**
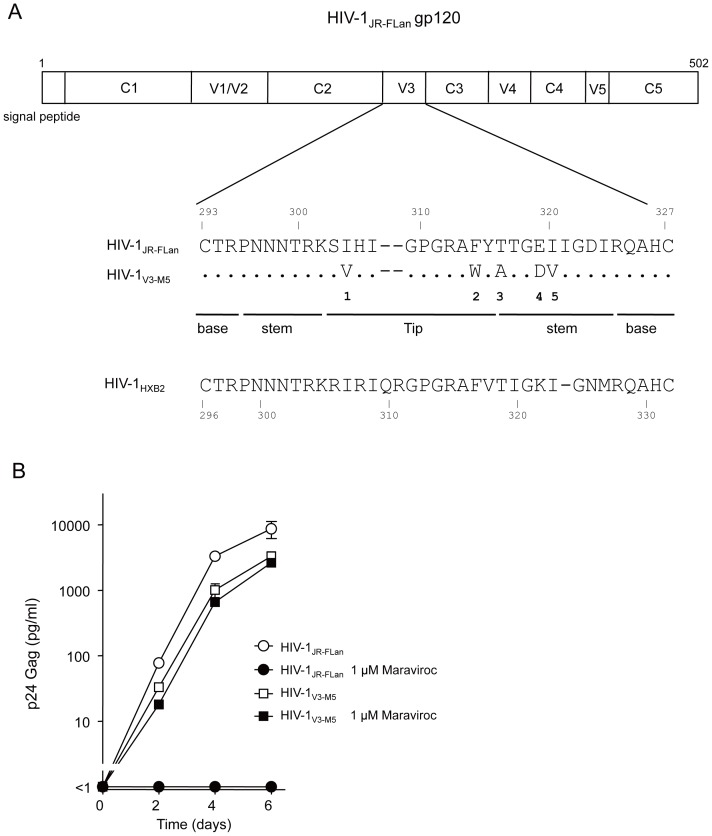
Noncompetitive resistant HIV-1_V3-M5_. (A) Five amino acid substitutions in the V3 loop of HIV-1_V3-M5_ (I304V/F312W/T314A/E317D/I318V). HIV-1_JR-FLan_ was created form HIV-1_JR-FL_ by incorporation of AflII and NheI. Incorporation of the NheI site led to amino acid substitutions Val^342^-Ile^343^ to Ala^342^-Ser^343^. HIV-1_JR-FLan_ was used as the parental virus. (B) Replication kinetics of HIV-1_V3-M5_ in the presence or absence of 1 µM maraviroc in PM1/CCR5 cells. PM1/CCR5 cells (1×10^5^) were infected with 10 ng of p24 Gag for 3 h. Viral replication was monitored by measuring p24 Gag in the supernatant after infection. The analysis was repeated three times; the error bars represent the S.D. of three replicates from one representative experiment.

HIV-1_V3-M5_ containing the five mutations I304V/F312W/T314A/E317D/I318V in the V3 loop with a JR-FL background (Figure 1A) exhibits noncompetitive resistance to maraviroc [15]. This virus could replicate in the presence of an extremely high concentration of the entry inhibitor (Figure 1B), i.e., 1 µM maraviroc, which was 147-fold higher than the IC_50_ value of the wild-type HIV-1_JR-FLan_ (0.0069 µM). HIV-1_V3-M5_ could infect PM1/CCR5 cells through drug-bound CCR5 to produce p24 Gag in the presence or absence of 1 µM maraviroc, whereas HIV-1_JR-FLan_ replication was completely suppressed.

### Suppression of replication in recombinant viruses containing one to three mutations in the V3 loop by maraviroc

**Figure 2:**
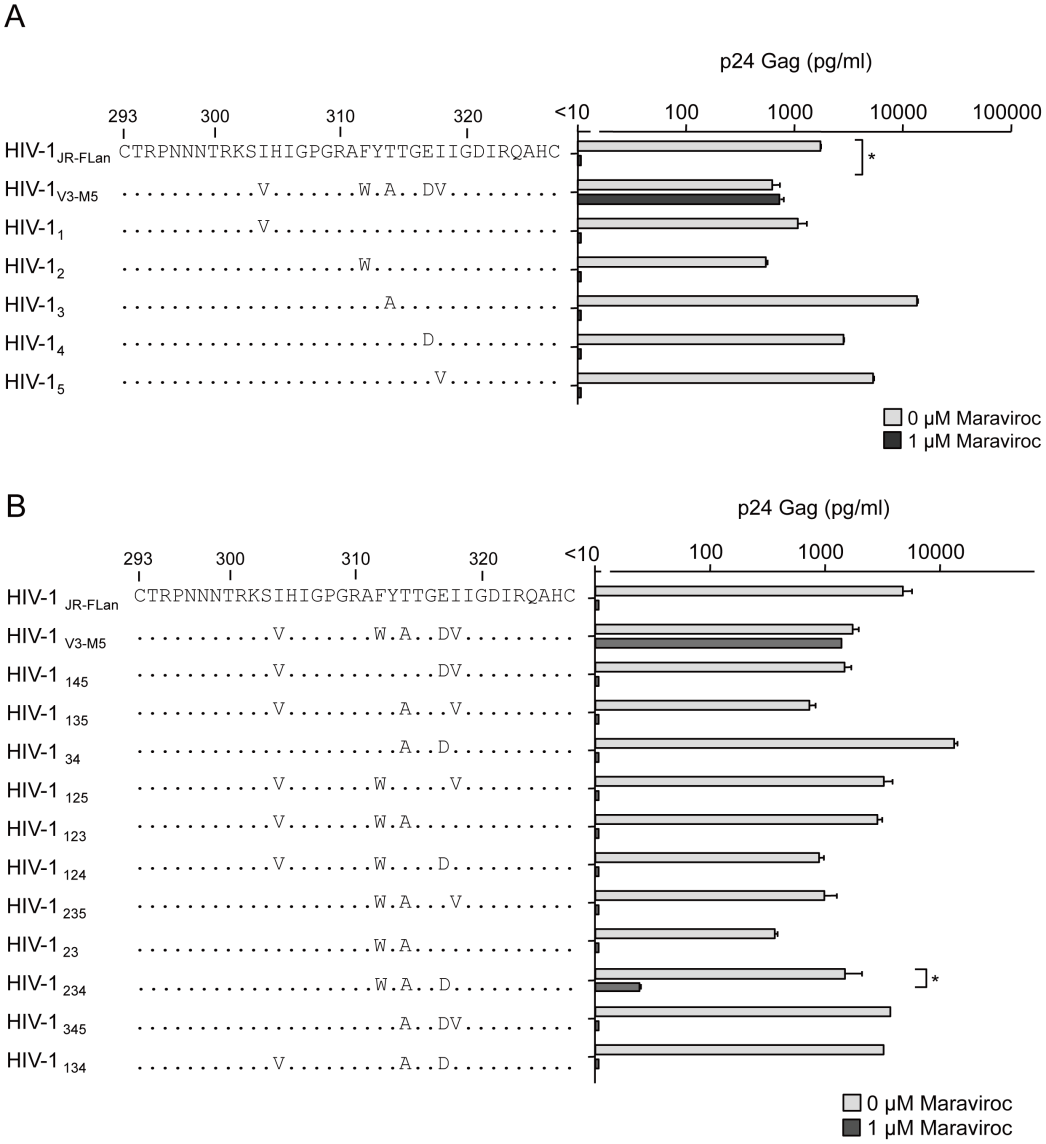
The effect of 1 µM of maraviroc on p24 Gag production in recombinant viruses containing one (A) and two or three (B) of the five amino acid substitutions. PM1/CCR5 cells (1×10^5^) were infected with 10 ng p24 Gag for 3 h in the presence or absence of 1 µM maraviroc. On day 6 after infection, the amount of Gag in the supernatant was measured using HIV-1 p24 ELISA. The analysis was repeated three times; the error bars represent the S.D. of three replicates from one representative experiment. **, *p*<0.01. Statistical significant difference was calculated by *t* test.

To further examine the contribution of each mutation to noncompetitive resistance, we constructed recombinant viruses containing one of the five mutations in the V3 loop (Figure 2A). I304V, F312W, T314A, E317D, and I318V were the polymorphic mutations detected in R5 clinical isolates. Thus, none of these viruses exhibited defective growth, although F312W caused a moderate decrease in p24 Gag production in the absence of maraviroc. HIV-1_V3-M5_ replication was 1.8-fold lower than HIV-1_JR-FLan_ replication. The presence of 1 µM maraviroc completely suppressed the production of recombinant viruses containing a single mutation, indicating that these single mutations could not confer noncompetitive resistance. Following this, we constructed 11 recombinant viruses, each containing two or three random combinations of the mutations (Figure 2B). Theoretically, the total number of possible combinations of the five mutations was 120; therefore, 11 combinations of two or three mutations were insufficient to determine the crucial combination(s) for noncompetitive resistance. These recombinants could produce more than 100 pg/ml p24 Gag in the absence of maraviroc, although their replication resulted in variable levels of p24 Gag. Maraviroc mostly suppressed the replication of these recombinant viruses, indicating that the combination of these two or three mutations did not confer use of drug-bound CCR5 as a coreceptor for viral entry. However HIV-1_234_ containing F312W/T314A/E317D could replicate in the presence of 1 µM maraviroc, although p24 Gag production was 1.8% of that in its absence. We could not passage HIV-1_234_ in PM1/CCR5 cells because of its poor replication in the presence of 1 µM maraviroc (data not shown). These results suggest that HIV-1_234_ is an intermediate form in the transition of the wild type to a completely noncompetitive-resistant form.

### Effect of maraviroc on recombinant viruses containing four mutations in the V3 loop

**Figure 3:**
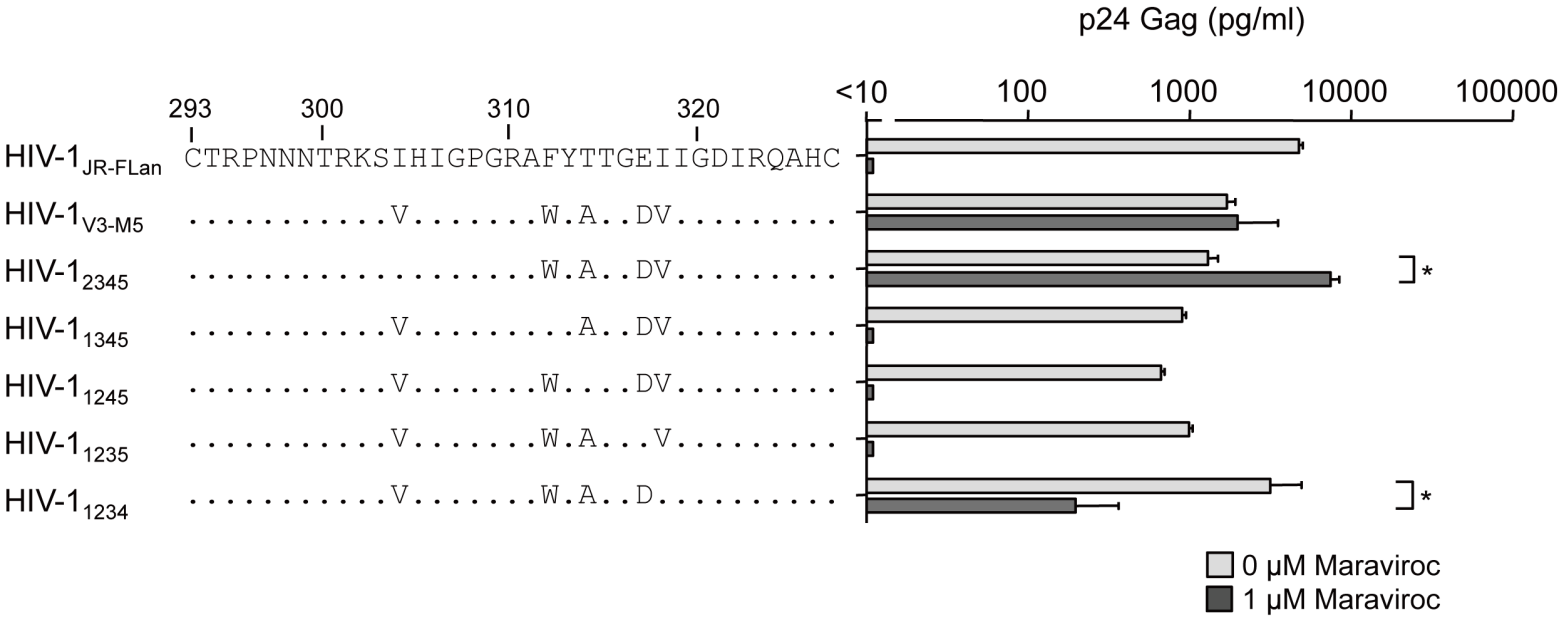
The effect of 1 µM of maraviroc on p24 Gag production in recombinant viruses containing four of the five amino acid substitutions. PM1/CCR5 cells (1×10^5^) were infected with 10 ng p24 Gag for 3 h in the presence or absence of 1 µM maraviroc. On day 6 after infection, the amount of Gag in the supernatant was measured using HIV-1 p24 ELISA. The analysis was repeated three times; the error bars represent the S.D. of three replicates from one representative experiment. **, *p*<0.01. Statistical significant difference was calculated by *t* test.

We next examined the recombinant viruses containing four mutations in the V3 loop (Figure 3). Without maraviroc, the viral fitness of HIV-1_1234_ was comparable with that of HIV-1_JR-FLan_, whereas the other four recombinant viruses replicated at levels lower than those of HIV-1_V3-M5_. Of note, HIV-1_2345_ and HIV-1_1234_ could replicate in the presence of 1 µM maraviroc, although HIV-1_1345_, HIV-1_1245_, and HIV-1_1235_ replication was completely suppressed. p24 Gag production by HIV-1_2345_ in the presence of maraviroc was 4.5-fold higher than that in its absence, whereas HIV-1_1234_ replication in the presence of maraviroc was 15-fold lower than that in the absence of maraviroc. These two viruses contained three common mutations: F312W, T314A, and E317D.

### Effect of maraviroc on recombinant virus containing F312W/T314A/E317D in the V3 loop

**Figure 4:**
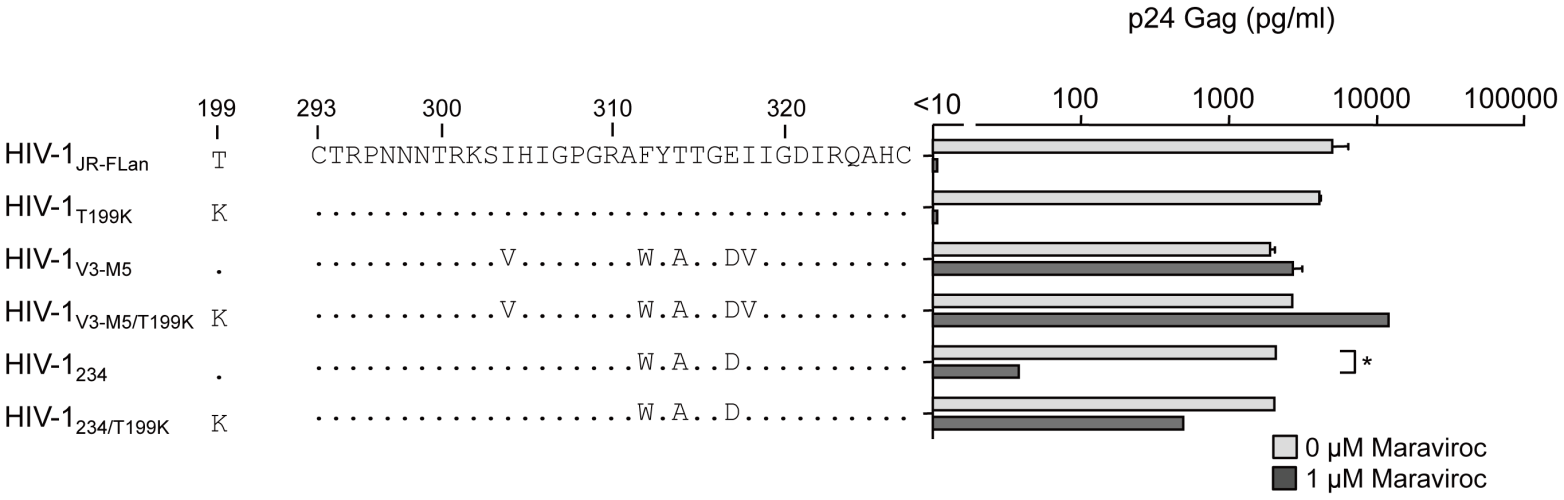
The effect of 1 µM of maraviroc on p24 Gag production in HIV-1_JR-FLan_, HIV-1_T199K_, HIV-1_V3-M5_, HIV-1_V3-M5/T199K_, HIV-1_234_, and HIV-1_234/T199K_. PM1/CCR5 cells (1×10^5^) were infected with 10 ng p24 Gag for 3 h in the presence or absence of 1 µM maraviroc. On day 6 after infection, the amount of Gag in the supernatant was measured using HIV-1 p24 ELISA. The analysis was repeated three times; the error bars represent the S.D. of three replicates from one representative experiment. **, *p*<0.01. Statistical significant difference was calculated by *t* test.

We further examined whether HIV-1_234_ containing the triplet mutation F312W/T314A/E317D exhibited noncompetitive resistance (Figure 4). HIV-1_V3-M5_ replication can be enhanced by T199K in V3 mutants to a level comparable with that in HIV-1_JR-FL_ [15]. p24 Gag production by HIV-1_V3-M5/T199K_ increased from 3100 pg/ml to 10,500 pg/ml in the presence of 1 µM maraviroc, whereas there was no significant increase in its absence. Similarly, HIV-1_234/T199K_ replication was significantly enhanced from 31 pg/ml to 650 pg/ml in the presence of 1 µM maraviroc but not in its absence. These results indicated that triplet mutations in the V3 loop are crucial for noncompetitive resistance, and I304V, I318V, or T199K can increase viral fitness.

**Figure 5:**
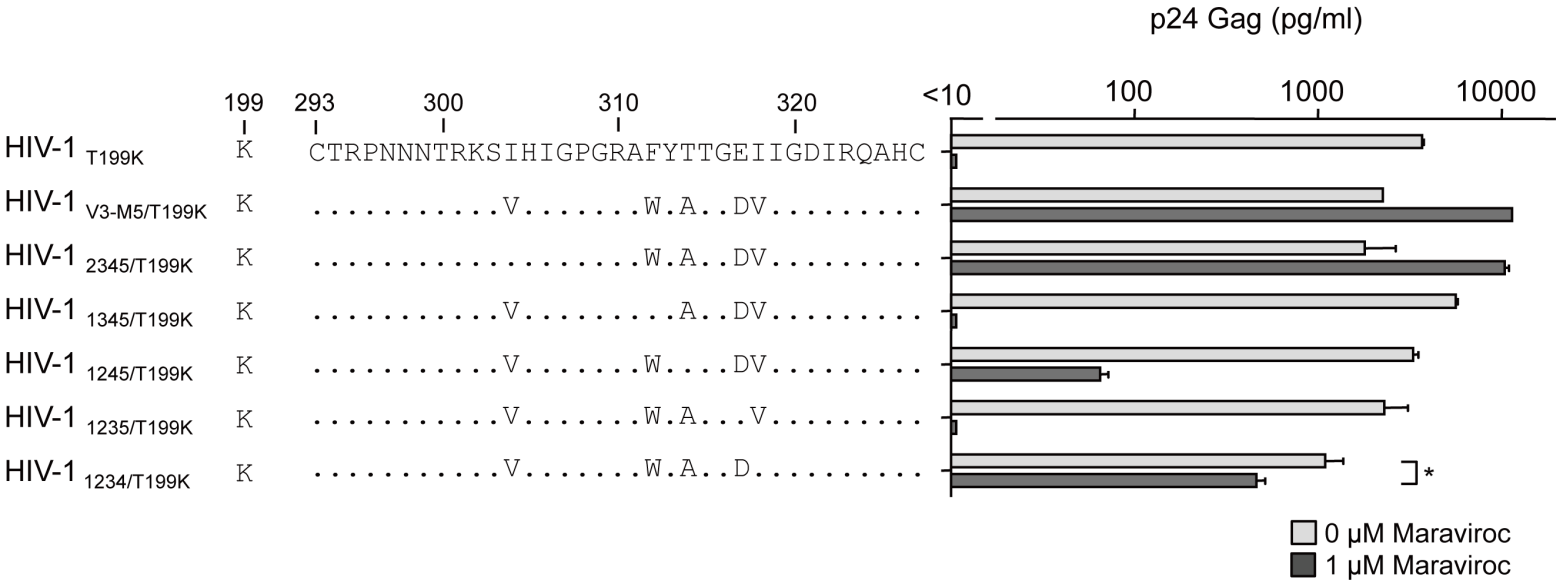
The effect of 1 µM of maraviroc on p24 Gag production in recombinant viruses containing four amino acid substitutions plus T199K. PM1/CCR5 cells (1×10^5^) were infected with 10 ng p24 Gag for 3 h in the presence or absence of 1 µM maraviroc. On day 6 after infection, the amount of Gag in the supernatant was measured by HIV-1 p24 ELISA. The analysis was repeated three times; the error bars represent the S.D. of three replicates from one representative experiment. *, *p*<0.05; **, p<0.01. Statistical significant difference was calculated by *t* test.

Finally, we examined the effects of T199K on the replication of recombinant viruses carrying four mutations in the V3 loop. In the presence of maraviroc, HIV-1_1234_ produced 6.7% of p24 Gag of that in its absence (Figure 3); however, HIV-1_1234/T199K_ replication increased up to 43% (Figure 5). Of note, HIV-1_1245_ replication was completely suppressed by 1 µM maraviroc (Figure 3); however, HIV-1_1245/T199K_ could replicate in the presence of 1 µM maraviroc, although the p24 production was only 2% of that in the absence maraviroc (Figure 5). These results indicated that the absence of T314A in the triplet could be compensated by I304V, I318V, or T199K and result in noncompetitive resistance.

### Susceptibilities of pseudotyped viruses containing four mutations in the V3 loop to maraviroc

**Figure 6:**
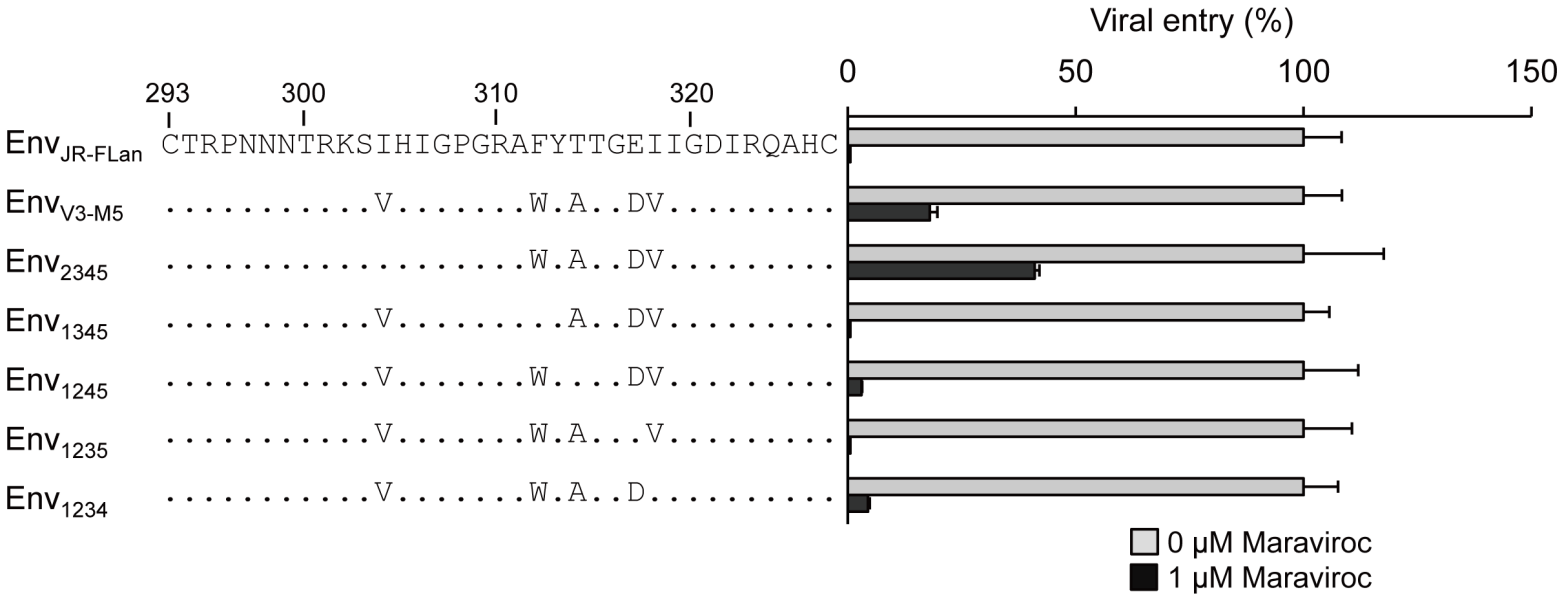
Maraviroc susceptibility of pseudotyped viruses derived from HIV-1_JR-FLan_, HIV-1_V3-M5_, HIV-1_2345_, and HIV-1_1345_, HIV-1_1245_, HIV-1_1235_, and HIV-1_1234_. MAGIC-5 cells were infected with pseudotyped viruses in the absence or presence of 1 µM maraviroc. The analysis was repeated three times; the error bars represent the S.D. of three replicates from one representative experiment. **, *p*<0.01. Statistical significant difference was calculated by *t* test.

To confirm the phenotypes of the recombinant viruses determined by the single-round infection assay using MAGIC-5 cells, we examined the susceptibility of viral entry using pseudotyped viruses with mutant envelopes (Figure 6). The viral entry of HIV-1_JR-FLan_ Env, HIV-1_1345_ Env, or HIV-1_1235_ Env was completely suppressed by maraviroc. These results were consistent with those obtained using competent viruses (Figure 3). HIV-1_V3-M5_ Env inhibition with maraviroc saturated approximately 17% entry efficiency [15]. HIV-1_1234_ Env retained 4% entry efficiency in the presence of 1 µM maraviroc, indicating that the low efficiency of drug-bound CCR5 usage accounted for the low replication rate of the competent virus. In contrast, HIV-1_2345_ Env could infect MAGIC-5 cells with 41% entry efficiency of that in the absence of maraviroc (Figure 6), although viral fitness in the presence of the inhibitor was superior to that in its absence in PM1/CCR5 cells (Figure 3). Furthermore, even 1 µM maraviroc did not completely suppress HIV-1_1245_ Env entry (Figure 6). These discrepancies may have occurred because of the cell-type-specific nature of noncompetitive resistance [34].

### MD simulations of the HIV-1 gp120 outer domain

**Figure 7:**
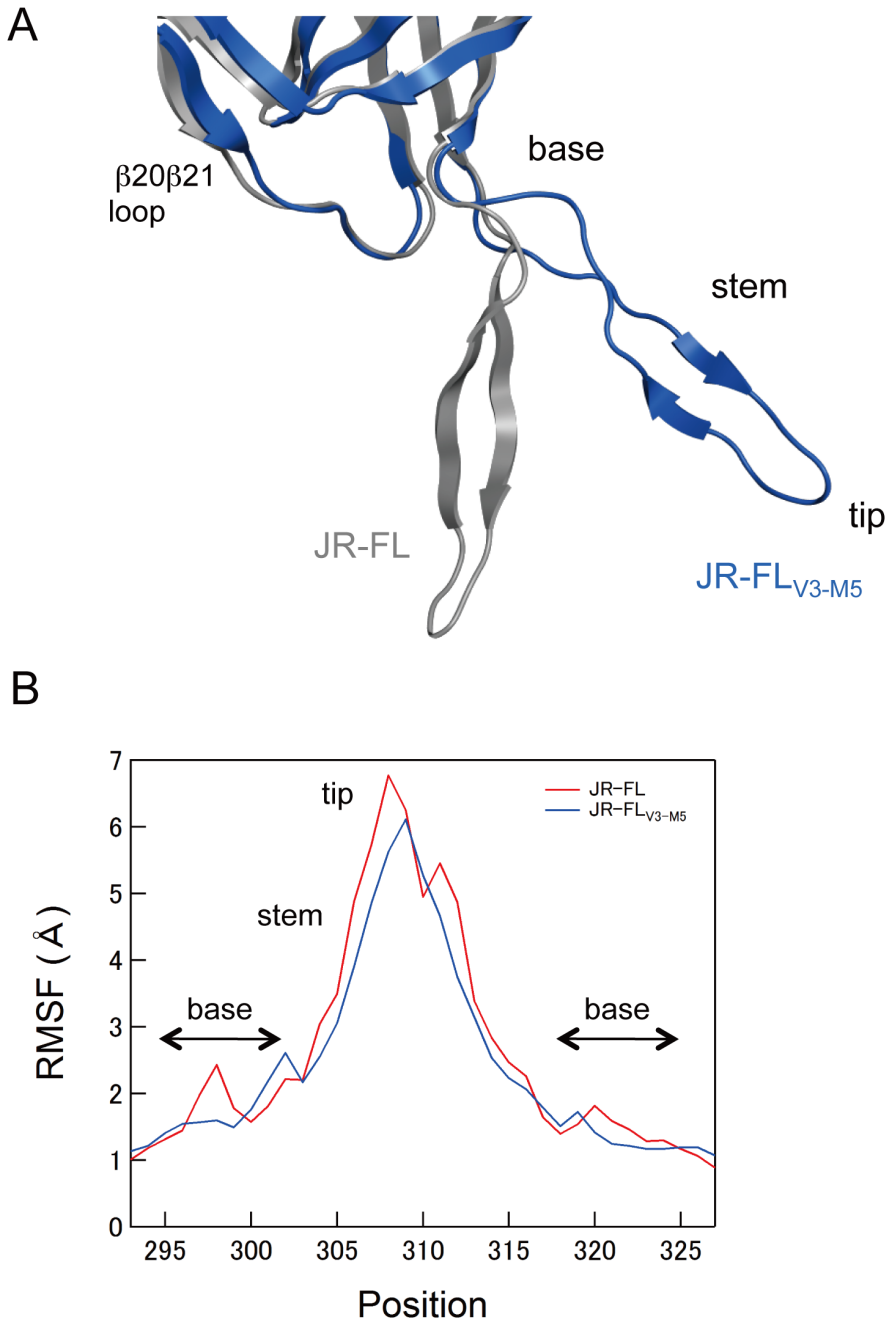
MD simulation of the HIV-1 gp120 outer domain. (A) Superimposition of averaged structures obtained from 40,000 snapshots during the 10–20 ns of MD simulation. Grey and blue ribbons indicate the gp120 V3 of JR-FL_an_ and JR-FL_V3-M5_, respectively. (B) Distribution of RMSF in the V3 region of gp120. The RMSF values indicate the atomic fluctuations of the main chains of individual amino acids during the 10–20 ns of MD simulations.

MD simulation is a powerful computational method for studying motions of proteins at the atomic-scale [23]–[25]. To address structural impacts of the V3 maraviroc-resistance mutations, we performed MD simulations of the HIV-1_JR-FL_ gp120 outer domain V3 loop with and without the five mutations of HIV-1_V3-M5_ (I304V/F312W/T314A/E317D/I318V). As described previously [21], [22], the root mean square deviation (RMSD) between the initial model and the model at a given time of MD simulation sharply increased soon after heating the initial model and then fluctuated continually for 20 ns of simulations (data not shown). The data suggests an intrinsic property of the gp120 outer domain V3 loops that results in structural fluctuations in solution. Hence, we constructed averaged gp120 structures using 40,000 snapshots during the 10–20 ns of MD simulation, and we superimposed them to reveal structural differences in the V3 loops of the two gp120s. Marked changes in V3 conformation were induced by introduction of the five V3 mutations (Figure 7A). The V3 loop of JR-FL_V3-M5_ was located at a much more distant position from the β20β21 loop in the outer domain than that of JR-FL. In addition, an anti-parallel β-sheet in the V3 stem region was reduced in the V3 loop of JR-FL_V3-M5_ compared with that of JR-FL.

To map the V3 loop sites in which fluctuations are influenced by the five mutations, we calculated the root mean square fluctuation (RMSF) of the main chains of individual amino acids in the V3 loop using 40,000 snapshots from 10–20 ns of each MD simulation (Figure 7B). The RMSF values were maximal at the V3 tip, indicating that the region involved in binding to CCR5 ECL2 fluctuates the most in solution. Interestingly, the five mutations were found to decrease the RMSF throughout the V3 tip and stem regions (Figure 7B, blue line). In addition, the five mutations caused a shift in small RMSF peaks at V3 base regions.

## Discussion

In this study, we examined the genetic and structural bases for the noncompetitive resistance of HIV-1 to maraviroc. Using site-directed mutagenesis, we demonstrated that combinations of mutations in V3 are required to confer maraviroc resistance to the HIV-1_JR-FL_ strain (Figures 2, 3). In addition, we showed that in combination with the V3 mutations, a T199K mutation in the C2 region enhanced viral fitness (Figures 4, 5). Finally, we indicated that these five maraviroc-resistance V3 mutations of HIV-1_V3-M5_ change the intrinsic structures and motion of the V3 loop on the HIV-1 gp120 outer domain. These data provide novel insights into the molecular mechanisms of HIV-1 maraviroc resistance. Further study may be able to classify the structure of V3 loop of HIV-1 to reveal or easily develop noncompetitive resistance through antiviral treatment with maraviroc in advance.

In the V3 loop, maraviroc-associated mutations have been reported at His^305^, Pro^308^, Ala^311^, Phe^312^, Thr^314^, Glu^317^, and Ile^318^ (numbering in JR-FL) [11], [35]–[37]. In the HIV-1_JR-FLan_ background, F312W/T314A/E317D is a crucial combination for maraviroc resistance, and I318V was required for extensive replication comparable with that in the wild type (Figure 5). HIV-1_234_ could not be passaged in PM1/CCR5 cells in the presence of 1 µM maraviroc because of its poor viral fitness (data not shown), suggesting that F312W/T314A/E317D is a type of fitness “valley” that needs to be selected on the genetic pathway for the development of noncompetitive resistance. F312W/T314A/E317D and one other mutation are required to acquire noncompetitive resistance. We could not select a maraviroc-resistant virus from the homogeneous viral population of HIV-1_JR-FLan_ because spontaneous multiple mutations (≧4) were unlikely to occur during *in vitro* passages, whereas our V3 virus library inherently contained F312W/T314A/E317D and fitness-enhancing mutations (I304V/I318V) [15]. We could not observe the condensation of viral clones containing one or two of these mutations at low concentrations of maraviroc (0.03–0.1 µM), suggesting that one or two combinations of these mutations did not confer a selective advantage (Figure 2). HIV-1 did not acquire maraviroc resistance by following a pathway for increasing resistance by the accumulation of multiple mutations. Instead, spontaneous alterations in the V3 loop were required to utilize maraviroc-bound CCR5. These results suggest that a virus library containing various mutations in specific regions such as the V3 loop is suitable for the *in vitro* selection of viruses resistant to entry inhibitors [38].

It remains unclear how the maraviroc resistant viruses use maraviroc-bound CCR5 as an entry coreceptor. Accumulating evidence from the investigations of protein chemistry indicates that structural fluctuations of the protein surface in solution play key roles in these molecular interactions [23]–[25]. Therefore, it is possible that the resistant viruses adjust these structural fluctuations of coreceptor binding surfaces through V3 mutations that enable binding to maraviroc-bound CCR5. In general, it is difficult to analyze motions of proteins at an atomic scale. However, recent advances in hardware and software of biomolecular simulation have rapidly improved its precision and performance [23]–[25]. Therefore, in this study we applied MD simulations and elucidated the structural dynamics of the gp120 outer domain in solution.

Our MD simulations of the gp120 outer domain suggest that the five mutations in the V3 loop of HIV-1_V3-M5_ caused marked changes in the physical properties of the CCR5 binding surface (Figure 7). Firstly, the mutations altered configurations and secondary structure of the tip-stem region of V3 loop on gp120. Secondly, the mutations reduced fluctuations at the base and tip regions of the V3 loop on gp120 and shifted the site of these fluctuations to the V3 base region. These results illustrate how maraviroc-resistance mutations have an impact on the intrinsic properties and structural motions of the V3 loops on the HIV-1 gp120 outer domain at the atomic-level. The altered configuration and/or fluctuation of the mutant V3 loops may advantageously support binding to drug-bound CCR5 by attenuating fluctuations on its surface. Further MD simulations in combination with experiments will clarify which of these structural changes are critical for the maraviroc resistance of HIV-1.
